# “I loved before, but now I love even more.” Qualitative study of posttraumatic growth as a consequence of severe COVID-19 experience in Slovak adults

**DOI:** 10.3389/fpsyg.2024.1335145

**Published:** 2024-02-20

**Authors:** Jana Tencerová, Peter Halama, Branislav Uhrecký

**Affiliations:** Institute of Experimental Psychology, Centre of Social and Psychological Sciences, Slovak Academy of Sciences, Bratislava, Slovakia

**Keywords:** thematic analysis, COVID-19, posttraumatic growth, adults, health psychology

## Abstract

**Introduction:**

The results indicate that post-traumatic growth does indeed occur after overcoming the severe form of COVID-19. It suggests that this posttraumatic growth most often occurred through a reassessment of priorities and an appreciation of life itself and loved ones. COVID-19 disease has been one of the most discussed and researched topics for several years, as it dramatically affects everyone’s daily life.

**Methods:**

The qualitative study presented here focuses on health psychology, especially post-traumatic growth after overcoming a severe form of the COVID-19 disease. We worked with adult people who had either been treated in the intensive care unit or had been hospitalized with severe pneumonia.

**Results:**

Thematic analysis was used to determine categories and subcategories. The study presented here contributes to knowledge about the COVID-19 experience by mapping a Slovakian sample of adult participants.

**Discussion:**

The results obtained by Thematic analysis help us better understand how people experience the disease, especially those who have overcome a severe form of the disease and thus had a borderline experience when their lives were directly threatened, as well as their overall health.

## COVID-19 and posttraumatic growth

The COVID-19 pandemic hit the world in early 2020, resulting in more than 700 million infections worldwide and causing more than 6 million deaths to date. In Slovakia, 1.8 million people have been infected to date and nearly 21,000 have succumbed to the disease [World Health Organization (n.d.)]. From a psychological perspective, the pandemic is considered a traumatic event because it has dramatically affected people’s daily lives in the form of disruption of normal routine activities, overall unpredictability of the situation, and loss of control. After the outbreak of the pandemic in Slovakia, security measures began to be implemented very quickly, restricting movement and contacts between people. As a result, people had to adapt to rapidly changing conditions with uncertain outcomes, while often being left to fend for themselves, with no social contact and support.

These negatives include, for example, reduced exercise and sedentary lifestyles, spending more time in front of a screen, and reduced social relationships and meetings with friends and colleagues (e.g., Brooks et al., 2020; Xiao et al., 2021). In addition to mental health issues, COVID-19 also negatively affects physical health. The most common, but less severe, consequences after experiencing the condition include, for example, limited ability to engage in physical activity (e.g., exercise) and musculoskeletal fatigue (Townsend et al., 2020; Shanbehzadeh et al., 2021). Other serious and common physiological consequences of overcoming this disease include, for example, sleep problems and insomnia (Garrigues et al., 2020), dyspnoea (Mandal et al., 2021), rheumatological symptoms (Halpin et al., 2021) and many others.

As mentioned at the outset, COVID-19 disease can be considered a collective trauma due to its wide-ranging impact (Masiero et al., 2020). The interconnection of mental and physical hardships only highlights how deeply this pandemic has affected people. However, the brighter side of the whole experience cannot be ignored, which in this case also manifested itself in people in the form of stress-related growth. The term was first proposed by Tedeschi and Calhoun (1996), who describe stress-related growth as a set of positive psychological changes that occur as a result of overcoming trauma or extremely challenging life circumstances. In contrast to the description of trauma and its effects on the human psyche and ultimately physicality, stress-related growth offers us an explanation of an extremely difficult situation from a positive perspective and thus forms an essential element for understanding the process of coping with trauma and its aftermath (Hyun et al., 2021; Waters et al., 2021). Research shows that this kind of growth is reflected in self-perception, interpersonal relationships, and general philosophy and attitude toward life (Tedeschi et al., 2014). It shows up for example in people who are dealing with serious health problems such as cancer (Casellas-Grau et al., 2017), HIV (Rzeszutek and Gruszczyńska, 2018). Gábor Maté, who became aware of an interesting fact while working in a palliative care ward, also reflects on this kind of growth after overcoming trauma. Some of the dying patients confided in him that their illness, although it would probably kill them in the end, was the best thing that could happen to them. They argued that it was the illness that showed them how inauthentic their lives had been and how far their perceived values had been from their true values (Maté, 2019). According to O’Leary and Ickovics (1995), this process can occur on multiple levels, each with its own quality and degree. The first, and lowest, level is *survival*. Those who merely survive the trauma have difficulty getting to their previous level of functioning in life. A slightly more positive development is *healing*, which, if it occurs, allows the person to function as effectively as before the trauma, but no significant change occurs. *Thriving*, as the third stage of post-traumatic development, represents the overcoming of the life lived before the trauma. The person who thrives from his or her suffering is getting beyond the level of his or her previous life, and it is the trauma experienced that helps him or her grow and develop. It is of course necessary to realize that traumatic events cannot be viewed only positively, as a precursor to growth. These experiences are deeply disturbing and wounding for a person. On the other hand, however, it is also important to realize that the psychological processes involved in coping with disruption are the same processes that can also bring about positive change and growth (Tedeschi and Calhoun, 2004).

### Current study

In this study we decided to qualitatively explore how participants reflected on posttraumatic growth after experiencing COVID-19 illness. As posttraumatic growth is usually identified after more serious stress events we chose the patients, who had experience the severe form of COVID-19 illness. The severe form was defined as being hospitalized during COVID-19 illness, either at Intensive Care Unit, or experiencing severe pneumonia. The main goal of the study was to describe a different manifestations of post-traumatic growth related to COVID-19. The study uses qualitative approach, specifically thematic analysis of participants statements. Every participant was interviewed by a researcher, with the questions explicitly directed to whether participant experienced the post-traumatic growth after COVID-19 and how it was manifested. In our paper, we adopted critical realistic position, which suppose that the data reflects experiences, meanings and the reality of participants, however, not in evident, unmediated fashion. These meanings and experiences are shaped by the broader social context which is also present in the data (Willig, 2012). In this study, we suppose, that the participants’ statements are shaped by general situation of the pandemic, and its media representations in the Slovak contexts.

It must be said that although COVID-19 is a new topic, there are already several studies that have investigated it in the context of post-traumatic growth, where it has also shown multiple manifestations in this direction (Asmundson et al., 2021), so it is safe to say that even such a complex and, in the case of our participants, life-threatening situation can lead to significant positive changes (Hyun et al., 2021). Moreover, it is possible that the worse the disease progresses and the more life-threatening it is, the more resources are mobilized in the person’s personality and the view of the tragedy changes to a more positive, meaninful event (Prieto-Ursúa and Jódar, 2020). In our design, we focused exclusively on a qualitative approach for two reasons. Firstly, we wanted to depict the individual and unique experiences of a person as reflected in their own words, in their own experience. Secondly, although the topic of COVID-19 is relatively new, its research has been approached predominantly quantitatively, due to the collection of large amounts of data in a short period of time. This approach is of course extremely beneficial, but the individual experience of a particular person is lost in the process. We used a fairly comprehensive interview method, covering the period before infection, during the acute phase of the disease and after discharge from hospital. To avoid researcher bias, we used a thematic analysis in which each researcher first created categories for themselves and then arrived at the final categories through repeated discussions and data editing.

## Method

### Participants

Participants were recruited based on three criteria: age over 18 years, a positive test for COVID-19, and hospitalization with a severe course of COVID-19 disease (either ICU stay or severe pneumonia). A total of 12 Slovaks (4 males and 8 females) aged 19–70 years of age (mean age 54.08 years) who had experienced a severe course of COVID-19 participated in the study. Participants were recruited using snowball method through several channels as hospitals, close acquaintances etc. We also wrote a mass e-mail to students with request to provide contacts to targeted persons. All participants signed an informed consent form and completed a demographic questionnaire prior to the study.

### Procedure

All participants gave their informed consent for inclusion before they participated in the study. The study was conducted in accordance with the Declaration of Helsinki, and the protocol was approved by the Ethics Committee [blinded for peer review]. The results are reported honestly and the study is not plagiarized.

After signing the informed consent, we arranged an interview date with the participant. Due to the demographic diversity and the poor epidemiological situation, the interviews were conducted exclusively online, using the Google Meet platform. The interview lasted approximately 1 h and the participant could end his/her participation at any time. Once the recording was obtained, the interview was transcribed for thematic analysis.

### Interview

The questions related to post-traumatic growth were part of the broader interview in which we mapped the participants’ personal experience of coping with the severe course of COVID-19 using a semi-structured interview, which had 4 major headings and a total of 36 questions. The first and second circles primarily mapped the person’s feelings and thoughts after the immediate onset of the disease and a description of how the disease broke out in a particular person and what the course was like. The third set of questions focused on the experience of hospitalization, treatment, and the evolution of the illness over time. The fourth heading, which also included the most questions, dealt with the period after discharge from hospital, current health and social life, family life, etc. For the purpose of this study, we processed answers from this part of the interview, where we directly asked respondents about aspects of positive changes as a result of the illness, focusing on personal development after surviving severe COVID-19 (e.g., Has anything changed for you and your life after you survived severe/critical COVID-19? Do you see yourself and your life differently now? What have you learned from this experience? What helps you in coping with the long-term consequences of the disease? Ask about coping resources, e.g., your personal qualities/the type of person you are, the fact that you survived, support from family and friends, availability of post-covid treatment, financial stability, etc.).

### Thematic analysis

The data from the interview were proceeded using thematic analysis (Braun and Clarke, 2006; Terry et al., 2018). Thematic analysis can be considered as a general qualitative method, which is focused on identification or examining the underlying ideas, assumptions, conceptualizations and/or ideologies that are theorized as shaping or informing the semantic content of the data. It involves the searching across a data set (in our case several interviews) to find repeated patterns of meaning, which can be interpreted as latent themes underlying the data.

In our study, we applied six steps suggested by Braun and Clarke (2006) to run thematic analysis. These steps include:

1. Familiarizing yourself with your data: this means transcription of the data and initial reading and/or re-reading the data, 2. Generating initial codes: this includes coding interesting features of the data in a systematic way across the entire data set, 3. Searching for themes: collating codes into potential themes, 4. Reviewing themes: Checking how the themes work in relation to the data and generating a thematic ‘map’ of the analysis. 5. Defining and naming themes: generating definitions and names for each theme. 6. Producing the report: Selection of examples for themes and producing a scholarly report of the analysis. However, the thematic analysis is not a strict linear process based on moving from one phase to the next, rather, it is considered to be a more iterative process, moving throughout the phases back and forth if needed (Terry et al., 2018).

The evaluation team consisted of three researchers, which are also authors of this study. All three researchers have prior experience with qualitative research in different settings, however, they have no prior research related to coping with COVID-19. The researchers had no presumptions about what could be the specific ways of posttraumatic growth in COVID-19 survivors, however, they believed that posttraumatic growth can happen in the context of COVID-19 illness. Prior to analysis, the researchers met and discussed the basic principles of thematic analysis to ensure that they would process the data in concordance with the method. Then, all researchers studied the data independently. Every researcher read the transcripts and identify the main topics in the data and their structure. Researchers wrote their themes downs and in the final step, all the researchers met again and repeatedly discussed the results with the emphasis on the differences between them. The differences were especially in names of themes, level of abstraction, structuring themes, and in the selection of the most representative quotes. For instance, two coders agreed on a theme of Phlegmatism (see Results), while one of the coders distinguished between Acceptance and Emotional distancing, which in the final results became its subthemes. The meetings were repeated until a consensus on the themes and its most proper representations was reached by all.

## Results

**Figure 1:**
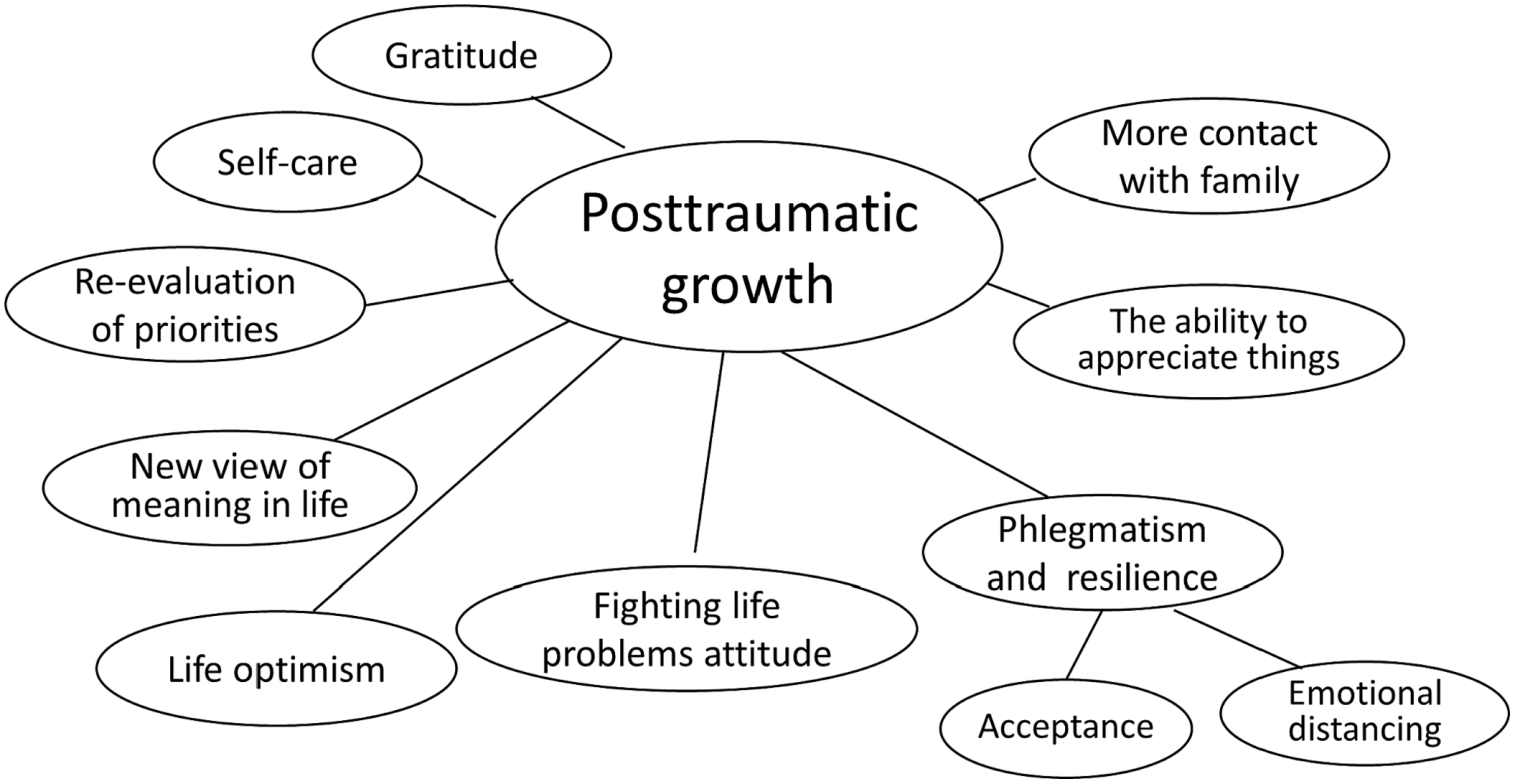
Themes of posttraumatic growth.

During the thematic analysis, we extracted 9 main themes or categories and two subthemes of posttraumatic growth after COVID-19 in a Slovak sample of participants (see Figure 1). Each of the themes is described lower in more details and themes with representative quotes are presented in the Table 1.

**Table 1:** Themes, subthemes and representative quotes.

Theme	Subtheme	Representative quote
Re-evaluation of priorities		“The priority is to go for a walk and we do not care that there is dust on the shelf. The important thing is to spend time together, or to visit somebody.”
New view of meaning in life		“Now I know, that it is wonderful to be alive. I feel like I’m alive a lot more than I was before. Everyone has only as much as they can carry.”
Life optimism		“I know I’ll be fine. This experience has not change my life for the worse.”
More contact with family		“I’m more excited about the family. Especially grandchildren. I want to see how they are doing, and how they’ll grow.”
Fighting life problems attitude		“I have gone through serious problems in the past. I knew that I would survive this.”
Phlegmatism and Resilience	Acceptance	“After this experience I am no longer afraid of death.”
	Emotional distancing	“Every bad period passes away and is replaced by a good one. This is how I set it in my mind and it helped me to overcome it.”
Gratitude		“I have seen a lot of dying in my life. I am all the more grateful that I survived and can always be here.”
Self-care		“It slowed me down. Now I make sure I do not forget to take good care of myself and my health.”
The ability to appreciate things		“When I was being discharged from the hospital, I noticed a small bird sitting next to the ambulance and I asked the paramedic to let me look at it for a while. I could not believe how beautiful he was.”

The first theme was *Re-evaluation of priorities*. The common denominator of this theme was the idea that what was a person’s main value before the illness, which guided the course of his/her life, was completely re-evaluated after the struggle for survival: “…only then did I realize that money played no role. Yes, they are important, but if I do not have health, I have nothing.” The second theme, *New view of meaning in life*, represented a fundamental reassessment of life goals and the question of what life really expects of us: “…I do not want to get in there and I do not want to do this job anymore. I knew that maybe I wanted to do something more meaningful. To search for some deeper meaning and simply pursue something that has a higher purpose. After all that I’ve been through.” This theme indicated that how one reflected on life and how one knew it before the illness had changed quite significantly, and that what previously made sense and was anchoring in one’s life had been altered to some extent. *Life optimism* was another theme and represented a form of positive attitude toward life that people found again after overcoming illness. The positive attitude toward life in this case meant that after overcoming the disease, people also saw a positive experience in the things and aspects of life that they had until recently considered negative. For example, one participant described the turn to a more optimistic experience as follows: “I did not have a good life until now, but now I’m going to enjoy it to the full.” *More contact with family* was also evident. Through the difficult course of the illness, people gained a new dimension in viewing their own family and loved ones: “I try, maybe even more, to appreciate every moment. With my family, with my husband that we have together. We do not argue – we argue 80% less than we used to on all the crap. Now we look at each other, smile, and tell each other that what we have been through is so much more than that crap about shoes being thrown and not put away, for example.” It was the situation in which they realized that they might not go back to their family, they might not even see their family anymore, that people fully understood its importance. The fact that visits to hospitals were forbidden throughout the critical period probably contributed to this fact. The only bridge of communication between the person in hospital and family members was telephone communication, with no possibility of real physical contact. Thus, support in a difficult moment was often provided by strangers who were part of the hospital staff (nurses, doctors, orderlies, etc.) The next theme, called *Fighting life problems attitude*, represented the strength gained through a difficult, uncertain situation. Characteristic in this case was an attitude where the person expressed determination and strength to cope with what in the past they would have considered insurmountable and unsolvable. Suddenly, these situations appear as a challenge driven by a positive expectation that the resources to cope are accessible and all will work out well in the end. The *Phlegmatism and resilience* theme had a similar quality. If something was a problem for a person in the past that they dealt with over and over again, now it is merely an annoyance on the periphery of survival: *“*If I was a phlegmatic before, I am even more so now. If something makes me angry now, it’s just such a mess to me, I try to get over it and realize that compared to what I’ve been through, it means nothing. I always tell myself that.” This theme was the only one that we further divided into two subcategories. The first subcategory was *acceptance*, which was a kind of surrender to life events, so that one no longer felt the need to control the circumstances of one’s life, but surrendered fully to what would come. The second subcategory was *Emotional detachment*, which stood for the realization that life is a sine curve and it is not possible to always be well. It was primarily about accepting the fact that there are good times and bad times in life, but that is normal and you can deal with it. The theme of *Gratitude* primarily referred to the fact that under the influence of this event, the person became fully aware of the gifts they had received in life, whether in the form of appreciation from family or simply being given more time in this world: “I realize that my time is limited, and I am all the more grateful that it has been granted to me. I will now decide to whom I will give my time and who is worthy of it.” The last theme was *Self-care*, the essence of which was to change habits (mental and physical) that served the psychological hygiene of the person, recognizing that he had not taken care of himself enough before: “When I feel really bad, I get dressed, take my backpack and go for a walk in nature. I stay there until I feel better and sort out my thoughts. Then it is necessary for me to be alone.”

## Discussion

The aim of the qualitative analysis above was to determine whether stress-related growth was a consequence of overcoming the severe form of COVID-19. We expected this growth to be mentioned in our data because several of the interview questions specifically referred to growth caused by stress and trauma.

Thematic analysis revealed that participants reflected on growth after overcoming the severe form of COVID-19 in the following main areas: Re-evaluation of priorities, New view of the meaning of life, Life optimism, More contact with family, Phlegmatism and resilience, Attitude toward fighting life problems, Gratitude and Self-care. Very similar results were obtained by Asmundson et al. (2021) in their study examining posttraumatic growth in patients with severe course of COVID-19. As in our analysis, the results showed that growth occurred most often through a greater appreciation of one’s own life, a greater appreciation of family and friends, an appreciation of everyday life, and a fundamental shift in priorities regarding what is truly important.

COVID-19 has been one of the most discussed but also most researched topics in recent years, mainly because of its high contagiousness, but also because of its high mortality rate. Research demonstrates that the COVID-19 pandemic has far-reaching consequences in people in the form of psychological depression and anxiety, but also a general deterioration of well-being (Vindegaard and Benros, 2020), decreased sleep quality (Huang and Zhao, 2020; Li et al., 2020), but also physical persistent complaints in the form of neurological complaints and dyspnea (Cares-Marambio et al., 2021), fatigue and joint pain (Carfì et al., 2020), or headaches (Davis et al., 2021).

In our study, we mainly focused on the positive aspect of overcoming the severe course of COVID-19 introduced by the so-called stress-related growth or post-traumatic growth, as it is a similar phenomenon. Basically, it involved the positive development of a person’s personality and characteristics after a borderline experience, often threatening death. In our study, the positive aspect of overcoming this illness proved to be a memorable part of this experience, generally manifesting itself in a fundamental change in the person’s priorities and personality attitudes. Previous research suggests that COVID-19 disease can also be a source of growth and positive experiences (Feng et al., 2021; Hyun et al., 2021; Waters et al., 2021). Our findings are partially consistent with the PTG scale, particularly the dimensions of more meaningful personal relationships and greater appreciation of life (Tedeschi and Calhoun, 2004). Although this division of the PTG is often cited, it is important to keep in mind that dimensional congruence for this growth is essentially nonexistent, as domains can vary considerably depending on cultural background and the diversity of traumatic events (Capielo Rosario et al., 2020). Therefore, it seems more appropriate to define growth after trauma as a combination of multiple aspects of human experience and the resulting benefits in cognitive, emotional, and social dimensions after struggling with traumatic events (Tedeschi and Blevins, 2015). This knowledge is consistent with our findings, as we can observe in the results of the thematic analysis the association between the cognitive (re-evaluation of priorities), emotional (ability to appreciate things), and social (more contact with family) levels of the participants’ reports. The study conducted with young people also shows that positive growth is possible and even quite common in the COVID-19 pandemic, with an important aspect of growth being a positive reassessment of one’s situation and an awareness of one’s strengths (Waters et al., 2021). In addition, we found that stress-related growth was also expressed in participants finding new meaning in life. In other words, people found a meaning in life after this difficult experience that they had not perceived before. According to the findings of Yıldırım and Arslan (2021), stress-related growth represents a moderator between the COVID-19 illness experience and meaning in life and is also positively related to life satisfaction. Similarly, Prieto-Ursúa and Jódar’s (2020) research suggests that positive growth is possible at multiple levels of human experience, indicating the potential for people to overcome challenges and use even a very difficult life experience, such as a severe, life-threatening illness, for their personal development. The positive growth described by our interviewees can be perceived in very similar ways, but we are also aware of the complexity of the moments these people had to live through. It may be that the more difficult the situation, the more a person’s inner resources are mobilized and their ability to transform tragedy into an opportunity or even a gift.

There are numerous studies that have looked in depth at posttraumatic growth or stress-induced growth and their association with COVID-19 disorders, but these variables have mainly been examined using quantitative methods (Tomaszek and Muchacka-Cymerman, 2020; Menculini et al., 2021; Vazquez et al., 2021; Özgüç et al., 2022). One of the major contributions of our analysis is that it explores this topic through interviews and captures the specific experiences of individuals with a severe course of illness. Another contribution is that we only invited individuals to participate in our study who had experienced a severe course of illness and were hospitalized (either on artificial respiration or with severe pneumonia). Therefore, we hypothesized that growth might be higher in individuals with this experience because they were exposed to a borderline situation in which death was imminent. For future studies, we recommend expanding the search for evidence on the psychological and physical consequences that this illness often entails, as these variables could also play an important role in growth. We also recommend expanding the number of participants and the age range between them.

## Data availability statement

The datasets presented in this article are not readily available because the original data contain sensitive information about the health status of the participants. The data are also available in Slovak language. Requests to access the datasets should be directed to janka.koroniova@gmail.com.

## Ethics statement

The studies involving humans were approved by the Ethical Committee of the Institute for Population and Human Studies – Bulgarian Academy of Sciences. The studies were conducted in accordance with the local legislation and institutional requirements. Written informed consent for participation was not required from the participants or the participants’ legal guardians/next of kin in accordance with the national legislation and institutional requirements. Written informed consent was obtained from the individual(s) for the publication of any potentially identifiable images or data included in this article.

## Author contributions

JT: Conceptualization, Formal analysis, Investigation, Writing – original draft, Writing – review & editing. PH: Formal analysis, Supervision, Writing – original draft, Writing – review & editing. BU: Conceptualization, Formal analysis, Writing – review & editing.
